# Gamma Interferon and Interleukin-17A Differentially Influence the Response of Human Macrophages and Neutrophils to Pseudomonas aeruginosa Infection

**DOI:** 10.1128/IAI.00814-18

**Published:** 2019-01-24

**Authors:** Sirina Muntaka, Yasir Almuhanna, Darryl Jackson, Sonali Singh, Afrakoma Afryie-Asante, Miguel Cámara, Luisa Martínez-Pomares

**Affiliations:** a School of Life Sciences, Faculty of Medicine and Health Sciences, University of Nottingham, Nottingham, United Kingdom; New York University School of Medicine

**Keywords:** gamma interferon, interleukin-1, interleukin-17, *Pseudomonas aeruginosa*, macrophages, neutrophils

## Abstract

Macrophages are important orchestrators of inflammation during bacterial infection, acting as both effector cells and regulators of neutrophil recruitment and life span. Differently activated macrophage populations with distinct inflammatory and microbicidal potentials have been described. Our previous work unveiled a positive and a negative correlation between levels of gamma interferon (IFN-γ) and interleukin-17A (IL-17A), respectively, and lung function in cystic fibrosis, particularly in patients chronically infected with Pseudomonas aeruginosa. This study sought to define key parameters in human antibacterial immunity under Th1- and Th17-dominated inflammatory conditions; the final aim was to identify unique characteristics that could be fine-tuned therapeutically to minimize tissue damage while maximizing bacterial clearance. Toward this aim, neutrophils were incorporated into cultures of macrophages treated with IFN-γ or IL-17A and infected with P. aeruginosa. The intent of this design was to model (i) initiation of inflammation by infected macrophages and (ii) delayed arrival of neutrophils and their exposure to macrophage-derived cytokines. Under these conditions, IFN-γ decreased bacterial killing and promoted the production of monocyte chemoattractant protein 1 (MCP-1). In contrast, IL-17A promoted bacterial killing but did not affect MCP-1 production. The level of secretion of the pyrogen IL-1β was significantly lower in the presence of IFN-γ than in the presence of IL-17A and correlated with levels of the *IL1B* transcript in infected macrophages. These findings support the validity of this model to investigate human antibacterial immunity. Based on these observations, the protective and damaging roles of IFN-γ and IL-17A, respectively, during P. aeruginosa infection could be caused by their contrasting effects on IL-1β and MCP-1 production.

## Abstract (precis)

Macrophages are important orchestrators of inflammation during bacterial infection, acting as both effector cells and regulators of neutrophil recruitment and life span. Differently activated macrophage populations with distinct inflammatory and microbicidal potentials have been described.

## INTRODUCTION

Macrophages and neutrophils are effective partners for a coordinated response to infection. Both cell types display complementary properties regarding life span, the production of antimicrobial products, the ability to degranulate and form extracellular traps, and the production of proinflammatory and anti-inflammatory cytokines and lipid mediators (1–4). There is increased evidence of a collaborative effort between both cell types in microbial clearance (1, 5–7). Nevertheless, the action of neutrophils during inflammation needs to be under strict control because of the undiscriminating toxicity of neutrophil products toward pathogens and host cells (3, 8).

Neutrophils are essential for protection against Pseudomonas aeruginosa *in vivo* (9) and can control P. aeruginosa infection *in vitro* through phagocytosis or NETosis (where NET refers to neutrophil extracellular traps) depending on the assay conditions (10). Macrophages recruit and potentiate the activity of neutrophils but can also trigger a “switch-off” response aimed at clearing the neutrophils and restoring organ function (2, 11). Similarly, macrophages are capable of responding to and incorporating neutrophil products, particularly myeloperoxidase, which will further activate macrophages (7). Thus, models enabling interactions between macrophages and neutrophils offer a physiologically relevant experimental setting to investigate what constitutes an effective immune response against P. aeruginosa, i.e., a response that improves neutrophil-mediated clearance of P. aeruginosa under controlled conditions so that tissue damage is minimized. Macrophages and neutrophils are both influenced by cytokines provided by adaptive immunity (12).

Our previous results showed that in cystic fibrosis (CF) patients, gamma interferon (IFN-γ) production by circulating immune cells positively correlated with lung function (13), while a negative correlation was found in the case of interleukin-17A (IL-17A) (13, 14). Both observations were particularly evident in CF patients chronically infected with P. aeruginosa (13). Human macrophages treated with IFN-γ failed to restrict P. aeruginosa growth, nor did they exhibit improved survival, upon infection but produced a different pattern of cytokines (13). In particular, IFN-γ promoted the synthesis of monocyte chemoattractant protein 1 (MCP-1) and increased tumor necrosis factor alpha (TNF-α)/IL-10 and IL-6/IL-10 ratios (13). We proposed that IFN-γ could be beneficial in CF by promoting a more balanced neutrophilic inflammatory response during infection that could protect against organ damage (13).

This work aimed to investigate the effect of Th1- and Th17-dominated inflammation on the cross talk between human macrophages and neutrophils during infection with P. aeruginosa to identify pathways that could influence infection outcome and the potential for inflammation and tissue damage. Th1 cell-mediated inflammation, dominated by IFN-γ, is generally associated with protection against intracellular pathogens (12). Th17 cells, through the release of IL-17A and granulocyte-macrophage colony-stimulating factor (GM-CSF), promote a neutrophilic inflammatory response best suited for clearance of extracellular bacteria and fungi (12).

To achieve this aim, macrophages were infected with P. aeruginosa, and after 2 h, neutrophils were incorporated into the cultures. The rationale behind this “macrophage-neutrophil stepwise coculture system” was to model the delayed arrival of neutrophils to the infection site and for the neutrophils to be exposed to cytokines produced by macrophages while enabling macrophage-neutrophil contact. Our previous results showed that P. aeruginosa-infected human macrophages were a major source of TNF-α, IL-6, IL-1β, IL-18, and IL-8, which will influence neutrophil function (13). To model Th1- and Th17A-dominated inflammation, macrophages were pretreated with either IFN-γ or IL-17A, and both cytokines were maintained throughout the assays.

Our results show that IFN-γ and IL-17A have contrasting effects on the responses of human macrophage-neutrophil stepwise cocultures to P. aeruginosa infection. Under our experimental conditions, IFN-γ reduced bacterial clearance by macrophage-neutrophil stepwise cocultures but had no direct effect on the ability of macrophage-only or neutrophil-only cultures to kill P. aeruginosa. IL-17A marginally increased bacterial killing by macrophage-neutrophil stepwise cocultures and had a direct effect on the ability of human neutrophils to kill P. aeruginosa. Neither cytokine altered the release of neutrophil elastase. In agreement with our previous findings, MCP-1 was selectively upregulated by IFN-γ (13). Differences between IFN-γ and IL-17A were observed regarding the level of production of IL-1β, which was significantly lower in IFN-γ- than in IL-17A-treated cultures. No qualitative differences in regard to cytokine production were observed between macrophage-only and macrophage-neutrophil cocultures under these experimental conditions. With the exception of IL-8, no cytokines were detected in supernatants from infected neutrophil-only cultures within the time frame of these assays. Modulation of IL-1β release by IFN-γ and IL-17A in macrophages correlated with changes in *IL1B* transcripts, suggesting that IFN-γ- and IL-17A-treated macrophages differ in their abilities to trigger *IL1B* transcription in response to P. aeruginosa infection. These observations provide novel insights into the proinflammatory and bactericidal potential of IFN-γ- and IL-17A-driven inflammation.

## RESULTS

### Effective killing of P. aeruginosa by neutrophils in the presence and absence of macrophages.

**FIG 1:**
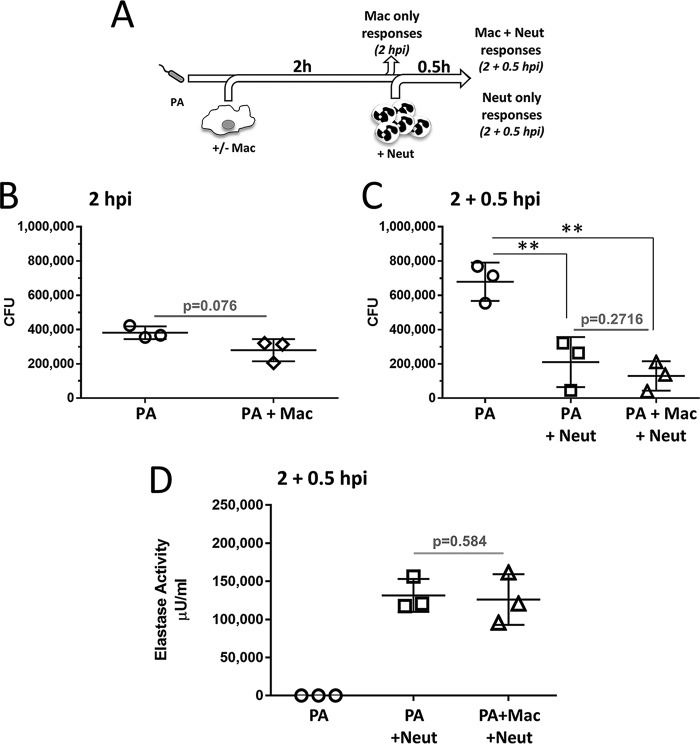
Bactericidal activity of neutrophils in the presence and absence of macrophages. (A) Schematic representation of the experimental setting. Human macrophages (Mac) (2 × 10^5^) generated in the presence of GM-CSF were infected with opsonized P. aeruginosa (PA) at an MOI of 0.5, as described in Materials and Methods. At 2 hpi, half of the culture was collected and processed for CFU quantification and preparation of supernatants, and freshly purified human neutrophils (Neut) were added (5 × 10^5^ neutrophils at a macrophage-to-neutrophil ratio of 1:5). Cocultures were incubated for 0.5 h and processed for CFU quantification and preparation of supernatants. (B) Minimal effect of macrophages on bacterial CFU (2 hpi). (C) Significant reduction in bacterial CFU by neutrophils in the absence and presence of macrophages (2 hpi plus 0.5 hpi). **, adjusted *P* < 0.01. (D) No differences in levels of neutrophil elastase in supernatants from infected macrophage-neutrophil cocultures and neutrophil-only cultures (2 hpi plus 0.5 hpi). No elastase activity was detected in the supernatants from cultures containing only P. aeruginosa (*n* = 3). Black lines, one-way ANOVA of P. aeruginosa, P. aeruginosa-neutrophil, and P. aeruginosa-macrophage-neutrophil samples; gray lines, paired two-tailed *t* test.

To model bacterial clearance during inflammation, i.e., initial exposure to macrophages followed by the arrival of neutrophils, opsonized P. aeruginosa was first cultured in the presence or absence of human macrophages, and at 2 h postinfection (hpi), neutrophils were added at a macrophage/neutrophil ratio of 1:5 (see Fig. S1 in the supplemental material for a detailed description of the assay). This experimental design (stepwise coculture) (Fig. 1A) incorporated sampling before the addition of the neutrophils to test for macrophage-only effects (i.e., “P. aeruginosa-only” and “P. aeruginosa-macrophage” samples). Cultures were terminated 0.5 h after the addition of neutrophils (i.e., P. aeruginosa-only, “P. aeruginosa-neutrophil,” and “P. aeruginosa-macrophage-neutrophil” samples). Samples were analyzed for CFU (2 h and 2 h plus 0.5 h) and neutrophil elastase (2 h plus 0.5 h). The bacterial load was marginally reduced by macrophages (samples at 2 hpi; *P* = 0.076) (Fig. 1B) but significantly reduced by the addition of neutrophils (2 h plus 0.5 h) (Fig. 1C). No clear additive effect between the bactericidal activities of macrophages and neutrophils was observed (samples at 2 h plus 0.5 h; *P* = 0.2716) (Fig. 1C). Experiments performed to test the contribution of neutrophils to the bactericidal activity of macrophage-neutrophil stepwise cocultures (i.e., to compare macrophage-neutrophil and macrophage-only cultures at 2 hpi plus 1 hpi) confirmed the dominant role of neutrophils in the bactericidal activity of the cocultures (Fig. S2). Supernatants from the cultures at 2 h plus 0.5 h were tested for the presence of neutrophil elastase, as described in Materials and Methods, as a means to investigate the effect of macrophages on the release of this enzyme. As shown in Fig. 1D, similar levels of neutrophil elastase were observed in the presence and absence of macrophages, indicating that macrophages do not affect elastase release. These results show that these experimental conditions enabled modeling of bacterial clearance during the sequential presence of macrophages and neutrophils and that neutrophils were effective in controlling P. aeruginosa infection independently of the presence of macrophages.

### Reduced control of P. aeruginosa infection by macrophage-neutrophil stepwise cocultures in the presence of IFN-γ.

**FIG 2:**
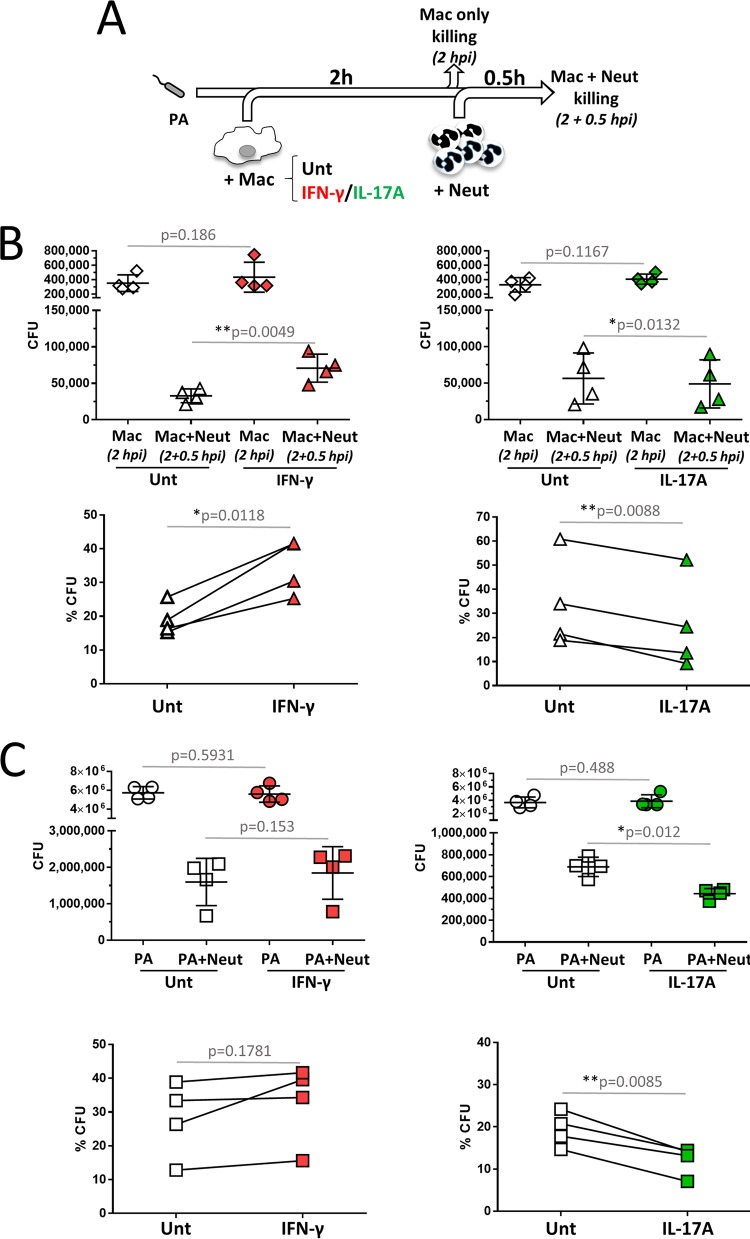
Effects of IFN-γ and IL-17A on the ability of macrophage-neutrophil stepwise cocultures to control P. aeruginosa growth. (A) Schematic representation of the experimental design. Human macrophages (2 × 10^5^) generated in the presence of GM-CSF were left untreated (Unt) or treated at day 6 with IFN-γ or IL-17A. On the following day, macrophages were collected and infected with P. aeruginosa at an MOI of 0.5 in the presence of human serum. At 2 hpi, half of the culture was collected and processed for CFU quantification, and freshly purified human neutrophils were added (5 × 10^5^ neutrophils at a macrophage-to-neutrophil ratio of 1:5). Cocultures were incubated for 0.5 h and processed for CFU quantification. (B) Effects of IFN-γ (left) and IL-17A (right) on the ability of macrophages and macrophage-neutrophil cocultures to control P. aeruginosa growth assessed as total CFU and as the percentage of P. aeruginosa bacteria remaining in the macrophage-neutrophil cocultures (2 hpi plus 0.5 hpi) compared to the macrophage-only cultures (2 hpi) (*n* = 4 for IL-17A and IFN-γ treatments). (C) Purified human neutrophils were pretreated for 0.5 h with either IFN-γ (left) or IL-17A (right) in the presence of GM-CSF and infected with P. aeruginosa at an MOI of 5 under opsonic conditions. Cytokines were maintained throughout. Cultures were processed for CFU analysis. IL-17A increased neutrophil-mediated killing of P. aeruginosa measured as total CFU and as the percentage of bacteria remaining compared to bacterium-only cultures. IFN-γ did not affect neutrophil-mediated killing of P. aeruginosa measured as total CFU and as the percentage of bacteria remaining compared to bacterium-only cultures. Neither IL-17A nor IFN-γ influenced P. aeruginosa growth (*n* = 4 for IL-17A and IFN-γ treatments). Gray lines, paired two-tailed *t* test.

In order to model the effects of Th1- and Th17-dominated responses on the capacity of these phagocyte stepwise cocultures to control bacterial infection, macrophages that were untreated or pretreated with IFN-γ or IL-17A for 24 h were infected with P. aeruginosa (multiplicity of infection [MOI] = 0.5) for 2 h, at which time half of the cultures were collected for analysis and neutrophils were added as described above. IFN-γ and IL-17A were maintained throughout the assay (Fig. 2A). Neither IFN-γ nor IL-17A affected the ability of macrophages to restrict P. aeruginosa infection (*P* = 0.186 for IFN-γ and *P* = 0.1167 for IL-17A by a paired *t* test) (Fig. 2B). IL-17A marginally promoted bacterial clearance by macrophage-neutrophil cocultures compared to untreated cultures (Fig. 2B), measured as total CFU (*P* = 0.0132 by a paired *t* test) and as the percentage of remaining bacteria (*P* = 0.0088 by a paired *t* test). In contrast, the reduction in bacterial counts in the cocultures in the presence of IFN-γ was significantly less pronounced than in untreated cultures, leading to an increased bacterial load measured as total CFU (*P* = 0.0049 by a paired *t* test) and as the percentage of remaining bacteria (*P* = 0.0118 by a paired *t* test) (Fig. 2B). To determine if IFN-γ and IL-17A could have a direct impact on the ability of human neutrophils to kill P. aeruginosa, neutrophils were pretreated with IFN-γ or IL-17A for 0.5 h and infected with P. aeruginosa at an MOI of 5. In agreement with the coculture results, IL-17A promoted bacterial clearance by neutrophils, assessed as total CFU (*P* = 0.012 by a paired *t* test) and the percentage of bacteria remaining (*P* = 0.0085 by a paired *t* test) (Fig. 2C). No effect of IFN-γ on the ability of neutrophils to control P. aeruginosa infection was detected when assessed as total CFU (*P* = 0.1530 by a paired *t* test) or as the percentage of bacteria remaining (*P* = 0.1781 by a paired *t* test) (Fig. 2C). P. aeruginosa growth was not directly affected by the presence of IL-17A or IFN-γ (Fig. 2C). Hence, IFN-γ reduced the clearance of P. aeruginosa by human macrophage-neutrophil stepwise cocultures. IL-17A slightly promoted P. aeruginosa clearance under these experimental conditions.

### IFN-γ and IL-17A do not influence release of neutrophil elastase in response to P. aeruginosa infection.

**FIG 3:**
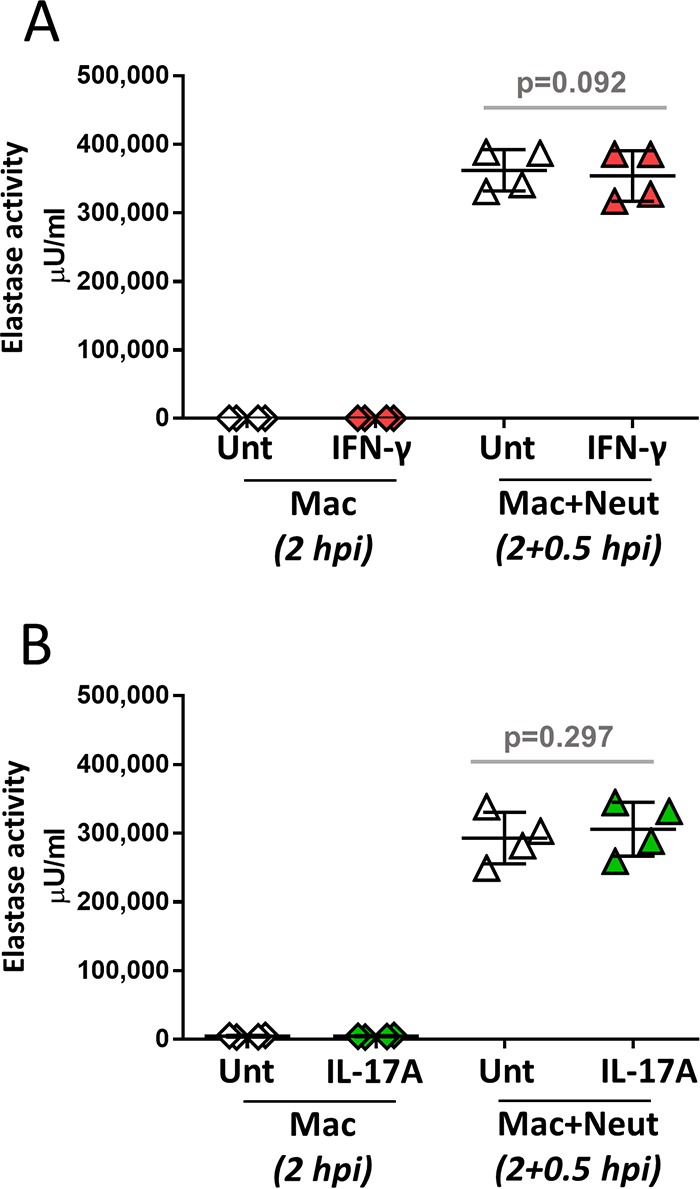
Release of neutrophil elastase by macrophage-neutrophil stepwise cocultures is not affected by IFN-γ (A) or IL-17A (B). Human macrophages (2 × 10^5^) generated in the presence of GM-CSF were left untreated or treated at day 6 with IFN-γ or IL-17A. On the following day, macrophages were collected and infected with P. aeruginosa at an MOI of 0.5 in the presence of human serum. At 2 hpi, half of the culture was collected and processed for CFU quantification and preparation of supernatants, and freshly purified human neutrophils were added (5 × 10^5^ neutrophils at a macrophage-to-neutrophil ratio of 1:5). Supernatants from macrophage-only cultures (2 hpi) and macrophage-neutrophil cocultures (2 hpi plus 0.5 hpi) were tested for the presence of neutrophil elastase as described in Materials and Methods (*n* = 4). Data were analyzed using a paired two-tailed *t* test.

Neutrophil elastase plays an important role in promoting tissue damage during inflammation. To determine if IFN-γ and IL-17A could modulate the release of neutrophil elastase in macrophage-neutrophil stepwise cocultures in response to P. aeruginosa infection, supernatants from infection assays described above (Fig. 2A) were analyzed for elastase activity (Fig. 3). As expected, elastase activity could not be detected in the supernatants from infected macrophages (2 hpi) in the presence or absence of cytokines but was readily observed in the neutrophil-containing cultures (2 h plus 0.5 hpi) and was not affected by the presence of IL-17A or IFN-γ.

### Differing effects of IFN-γ and IL-17A on cytokine production in response to P. aeruginosa.

**FIG 4:**
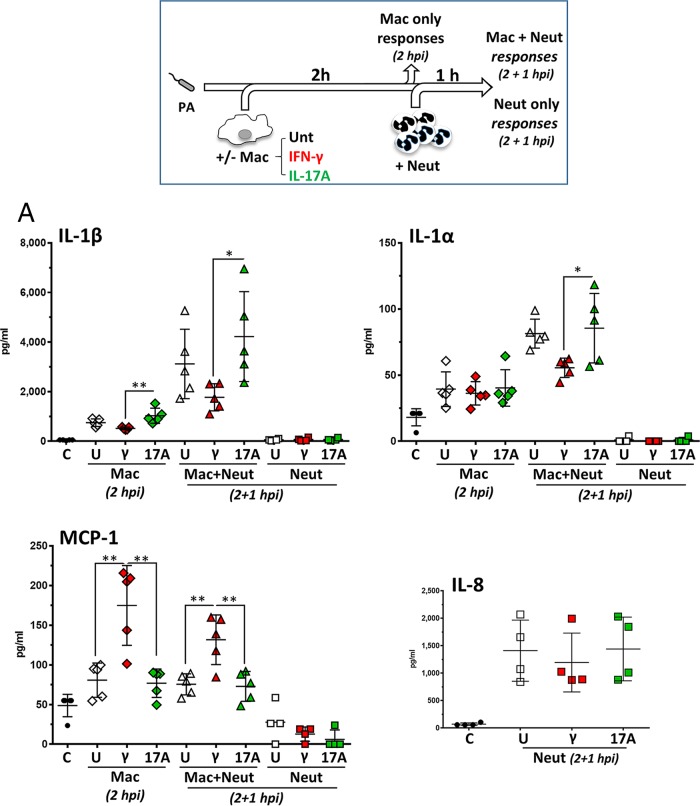

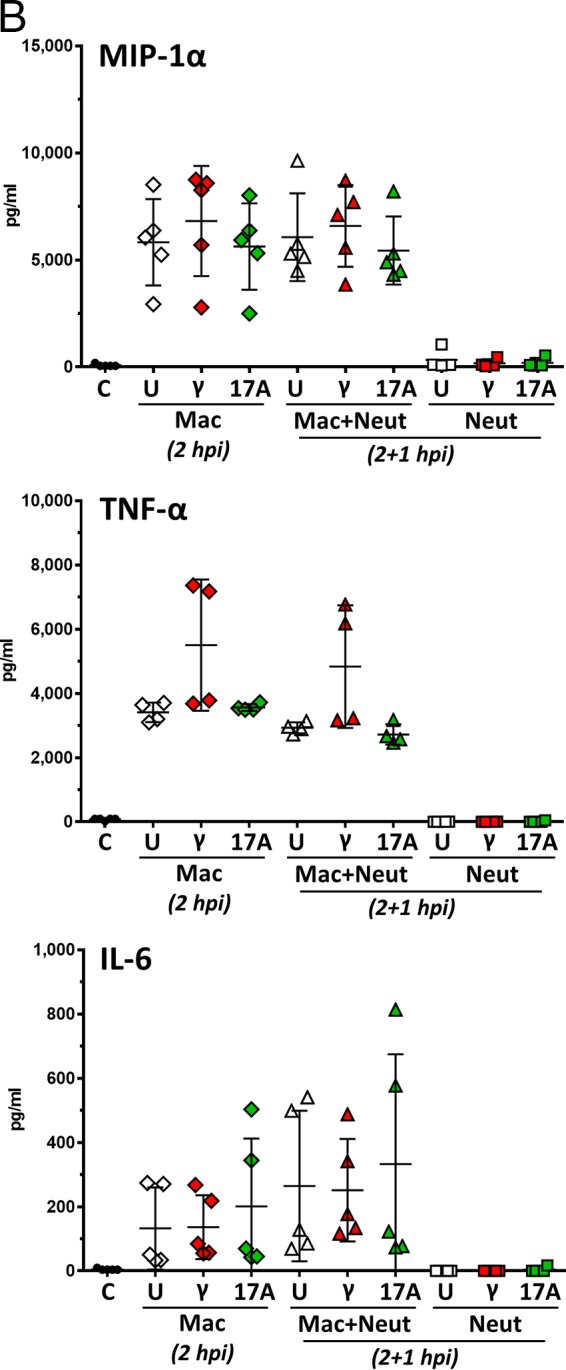
IFN-γ and IL-17A regulate IL-1β and MCP-1 production by macrophage-neutrophil stepwise cocultures in response to P. aeruginosa infection. Human macrophages (2 × 10^5^) generated in the presence of GM-CSF were left untreated or treated at day 6 with IL-17A or IFN-γ. On the following day, macrophages were collected and infected with P. aeruginosa at an MOI of 0.5 in the presence of human serum. At 2 hpi, half of the culture was collected and processed for preparation of supernatants, and freshly purified human neutrophils were added to the macrophage cultures (5 × 10^5^ neutrophils at a macrophage-to-neutrophil ratio of 1:5). Cocultures were then incubated for 1 h and processed for preparation of supernatants. (A) IFN-γ specifically upregulates MCP-1 synthesis compared to untreated and IL-17A-treated cultures. The levels of production of IL-1β were significantly different under IFN-γ and IL-17A conditions, being lower in the presence of IFN-γ and higher in the presence of IL-17A. Cultures lacking macrophages collected at 2 h plus 1 h were also investigated (Neut only) and produced IL-8 (bottom right). **, adjusted *P* < 0.01; *, adjusted *P* < 0.05. (B) Levels of MIP-1α, TNF-α, and IL-6 are unaffected by IFN-γ or IL-17A. C, medium only; U, untreated; γ, IFN-γ treated; 17A, IL-17A treated (*n* = 5).

To determine the effects of IFN-γ and IL-17A on the ability of human phagocyte stepwise cocultures to promote inflammation during P. aeruginosa infection, macrophages and neutrophils were infected as described above in the presence and absence of IFN-γ or IL-17A (Fig. 4), and supernatants were collected and analyzed for the presence of IL-1β, IL-1α, MCP-1, IL-8 (Fig. 4A), MIP-1α (macrophage inflammatory protein 1α), TNF-α, and IL-6 (Fig. 4B). In this instance, neutrophil-only cultures were also investigated by performing parallel assays without macrophages. The production of IL-1β, IL-1α, TNF-α, and IL-6 was completely dependent on bacterial infection, but low levels of MCP-1 and MIP-1α were already detected in the supernatants from uninfected cultures (see Fig. S3 in the supplemental material). IL-8 was detected under all conditions, but levels were above the range of the assay in all cultures containing macrophages. IL-8 was the only cytokine that could be clearly detected in neutrophil-only cultures and was not affected by the presence of IFN-γ or IL-17A (Fig. 4A, bottom right). Macrophages infected with P. aeruginosa (2 hpi) produced all cytokines tested, and no significant differences between macrophages that were left untreated and those that were treated with IL-17A were observed (Fig. 4A and B). IFN-γ-treated macrophages produced increased levels of MCP-1 and less IL-1β than untreated macrophages (significant only for MCP-1 [*P* = 0.002]) or macrophages treated with IL-17A (significant for MCP-1 [*P* = 0.0015] and IL-1β [*P* = 0.0043]) (Fig. 4A). These differences were maintained after the addition of neutrophils (2 hpi plus 1 hpi), with IL-1β production being significantly lower in IFN-γ-treated cocultures than in those treated with IL-17A (*P* = 0.0362) and MCP-1 production being significantly increased compared to untreated (*P* = 0.0049) and IL-17A-treated (*P* = 0.0036) cultures (Fig. 4A). Levels of IL-1α were low in all instances, but IFN-γ also showed a negative effect on IL-1α release by macrophage-neutrophil cocultures compared to IL-17A-treated samples (*P* = 0.04). Levels of MIP-1α, TNF-α, and IL-6 were not affected by IFN-γ or IL-17A (Fig. 4B). These observations indicate that IFN-γ targeted the ability of human phagocytes to regulate inflammation by reducing the release of IL-1β and IL-1α, particularly in contrast to IL-17A, while increasing the release of MCP-1.

### IL-1β production is caspase mediated during infection of human phagocyte stepwise cocultures with P. aeruginosa.

**FIG 5:**
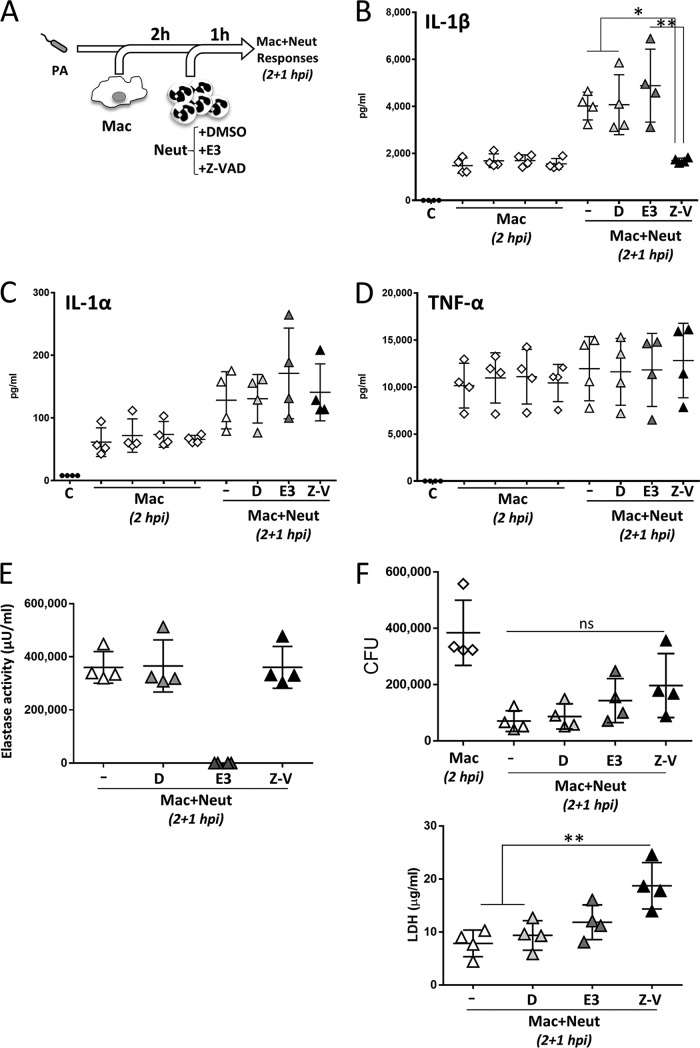
IL-1β production by infected macrophage-neutrophil cocultures is caspase dependent. (A) Schematic representation of the experimental design. Untreated human macrophages (2 × 10^5^) generated in the presence of GM-CSF were collected and infected with P. aeruginosa at an MOI of 0.5 in the presence of human serum. At 2 hpi, half of the cultures were collected and processed for preparation of supernatants and CFU quantification, and freshly purified human neutrophils were added (5 × 10^5^ neutrophils at a macrophage-to-neutrophil ratio of 1:5) in the absence and presence of dimethyl sulfoxide (DMSO) (D), neutrophil elastase inhibitor III (E3), or the pancaspase inhibitor Z-VAD (Z-V). Cultures were incubated for 1 h and processed for CFU and preparation of supernatants. (B) IL-1β production by infected macrophage-neutrophil cocultures is reduced by the caspase inhibitor but is unaffected by the elastase inhibitor. (C and D) Neutrophil elastase and caspase activities do not contribute to IL-1α and TNF-α production by infected macrophage-neutrophil stepwise cocultures. (E) The presence of Z-VAD does not affect elastase activity during infection. (F) Effects of elastase and caspase inhibitors on bacterial killing and cytotoxicity (LDH release) in macrophage-neutrophil stepwise cocultures. C, medium only (*n* = 4). Data were analyzed using one-way ANOVA with Tukey’s posttest. **, adjusted *P* < 0.01; *, adjusted *P* < 0.05.

P. aeruginosa has been shown to trigger IL-1β processing through the NLRC4 inflammasome (15), and Patankar et al. demonstrated that IL-1β production after pulmonary and peritoneal infection with P. aeruginosa was dependent on neutrophils and caspase-1 but independent of apoptosis-associated speck-like protein containing a caspase recruitment domain (ASC) (16). Proteases derived from neutrophils and mast cells have been implicated in IL-1β processing, and this is considered a way to amplify inflammatory responses (17). Karmakar et al., using a model of corneal infection, demonstrated IL-1β production in the absence of NLRC4 and ASC and indicated a role for neutrophil serine proteases in IL-1β processing in mouse and human neutrophils in response to P. aeruginosa infection (18). To investigate if neutrophil elastase, a serine proteinase, could contribute to the generation of IL-1β during the period of macrophage-neutrophil coculture and to test the contribution of caspases to this process, infections in which inhibitors of elastase or caspases were incorporated at the time of neutrophil addition were performed (Fig. 5A). The results demonstrated that IL-1β release by macrophage-neutrophil cocultures was dependent on caspase activity (Fig. 5B), as it was drastically reduced by the pancaspase inhibitor Z-VAD [*N*-benzyloxycarbonyl-Val-Ala-Asp(O-Me) fluoromethyl ketone] and not affected by inhibition of neutrophil elastase. Levels of IL-1α and TNF-α were not affected by the presence of either inhibitor (Fig. 5C and D). Comparison of cytokine levels between macrophage-neutrophil and macrophage-only infected cultures at 2 hpi plus 1 hpi confirmed that neutrophils do not promote cytokine production in the cocultures (see Fig. S4 in the supplemental material). Analysis of neutrophil elastase activity in supernatants confirmed the effectiveness of the inhibitor under our experimental conditions and that Z-VAD did not affect elastase activity (Fig. 5E). Both inhibitors appeared to promote bacterial growth during infection, but this effect did not reach significance (Fig. 5F, top). This observation correlated with the cell survival analysis based on lactate dehydrogenase (LDH) release, which indicated increased cell lysis in the presence of the elastase inhibitor and Z-VAD (Fig. 5F, bottom), with this difference becoming significant when comparing cultures lacking inhibitors and those treated with Z-VAD. This observation could be caused by the induction of necroptosis in neutrophils in the presence of apoptosis inhibitors. Necroptosis has been described to occur with Z-VAD treatment in the presence of TNF-α which is produced under our assay conditions (19). The reduction in IL-1β production by the pancaspase inhibitor Z-VAD but not by the elastase inhibitor indicates that elastase is not required under our experimental conditions and supports caspase-dependent processing of pro-IL-1β during infection of macrophage-neutrophil stepwise cocultures with P. aeruginosa.

### Transcription of *IL1B* in human macrophages in response to P. aeruginosa infection is reduced by IFN-γ and promoted by IL-17A.

**FIG 6:**
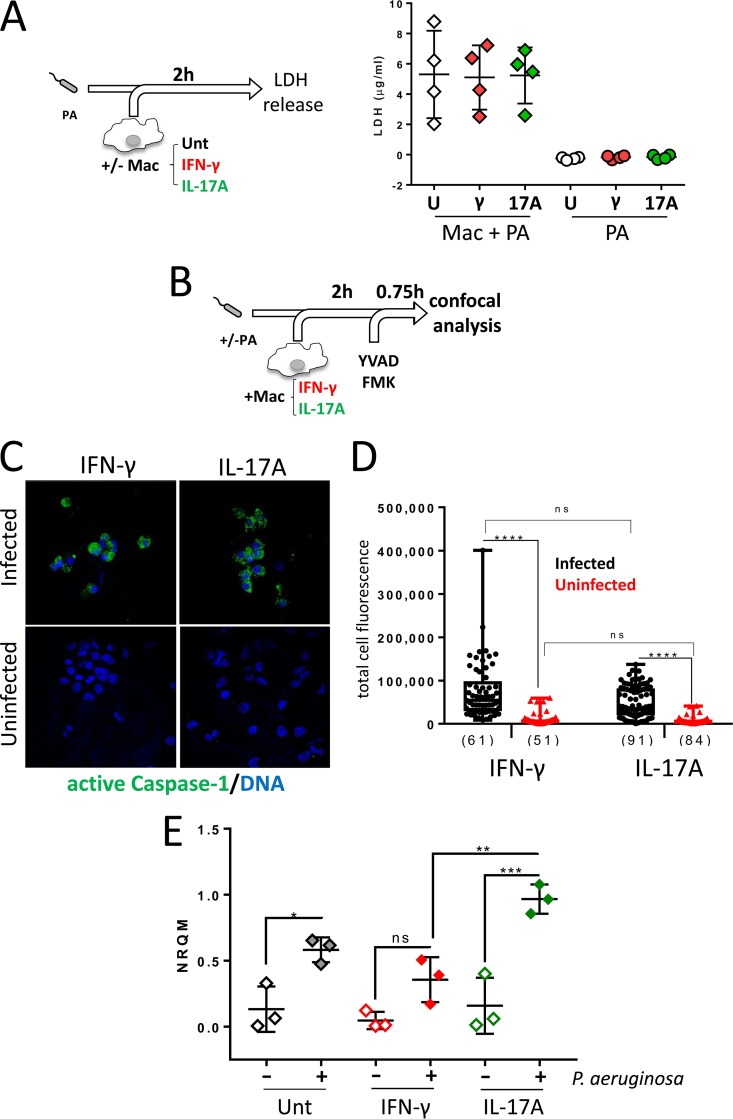
IFN-γ and IL-17A influence IL-1β production by P. aeruginosa-infected macrophages at the level of transcription. (A, left) Schematic representation of the experimental design to investigate LDH release. (Right) Similar LDH activity in supernatants from untreated (U) and IFN-γ (γ)- or IL-17A (17A)-treated macrophages in response to P. aeruginosa infection. (B) Schematic representation of the experimental design to investigate caspase-1 activation in macrophages. Infected and uninfected IFN-γ- or IL-17A-treated macrophages were treated at 2 hpi with Fam-flica for 45 min and processed for confocal analysis. (C and D) Comparable caspase-1 activation in IFN-γ- and IL-17A-treated macrophages upon infection with P. aeruginosa. (C) Representative z-stack images of infected and uninfected macrophages. Green fluorescence due to caspase-1 activation was detected in infected cultures but was not affected by the cytokine treatment. (D) Quantification of total cell fluorescence shows specific increases upon infection but no differences between IFN-γ- and IL-17A-treated samples. Numbers of cells used to generate the graph are shown. Data were collected from 3 independent repeats, and an average of 17 to 30 cells per repeat were assessed. Significance was determined using a Kruskal-Wallis test with Dunn’s posttest. (E) Analysis of *IL1B*-specific mRNA in untreated and IL-17A- and IFN-γ-treated macrophages upon infection with P. aeruginosa. IFN-γ- and IL-17A-treated and untreated macrophages were infected with P. aeruginosa as described in Materials and Methods. Samples were collected at 0.5 hpi and processed for qPCR to investigate levels of the *IL1B* transcript. Data are from three independent biological replicates, with each having three technical repeats. Significance was determined using one-way ANOVA with Tukey’s posttest. ns, not significant. ****, adjusted *P* < 0.0001; ***, adjusted *P* < 0.001; **, adjusted *P* < 0.01; *, adjusted *P* < 0.05. NRQM, normalized quantity mean.

Next, the mechanism by which IFN-γ and IL-17A caused such contrasting effects on IL-1β release by human macrophages in response to P. aeruginosa (Fig. 4A, IL1β graph) was investigated. Changes in IL-1β production suggested the existence of differential activation of inflammasomes under IFN-γ and IL-17A conditions. To investigate this possibility, supernatants from infected macrophages were tested for LDH activity (Fig. 6A), as inflammasome activation is generally linked to an inflammatory form of cell death termed pyroptosis; pyroptosis will lead to increased LDH release. Similar LDH levels were detected between IFN-γ- and IL-17A-treated cultures (Fig. 6A), indicating that differences in inflammasome activation might not be responsible for these observations. To further support these findings, caspase-1 activity in infected IFN-γ- and IL-17A-treated macrophages was investigated using the active caspase-1-specific label Fam-flica (FAM-YVAD-FMK). In brief, macrophages treated with IFN-γ or IL-17A were infected with P. aeruginosa, and Fam-flica was added at 2 hpi for 45 min (Fig. 6B). Cells were then collected and processed for confocal microscopy (Fig. 6C and D). Infected cells, in contrast to uninfected cells, readily became Fam-flica positive, indicating that caspase-1 activation was dependent on infection (Fig. 6C). The z-stack projections showed active caspase-1 distributed throughout the macrophage cytoplasm rather than being concentrated in single speck-like structures, which could be caused by the experimental conditions used in this study. Analysis of the total cell fluorescence intensity (Fig. 6D) and the percentage of infected cells containing active caspase-1 (see Fig. S5 in the supplemental material) indicated similar caspase-1 activities in the IFN-γ- and IL-17A-treated cultures, which further suggests that the levels of inflammasome activation are comparable under both conditions. Because IL-1β production is a two-step process first requiring the induction of pro-IL-1β synthesis, we next determined if the differences in secreted IL-1β levels were caused by changes in *IL1B* transcription. For this, untreated and IFN-γ- and IL-17A-treated macrophages were infected with P. aeruginosa for 0.5 h and processed for quantitative PCR (qPCR) analysis (Fig. 6E). *IL1B* transcription in human macrophages was upregulated by infection, and in agreement with the IL-1β secretion data, a trend was observed by which IFN-γ reduced and IL-17A increased *IL1B* transcription compared to the levels in untreated macrophages; differences between the IFN-γ and IL-17A conditions reached significance. These results support the differential abilities of IFN-γ- and IL-17A-treated macrophages to trigger *IL1B* transcription in response to P. aeruginosa infection.

## DISCUSSION

The main findings of this study can be summarized as follows: (i) neutrophil-mediated killing of P. aeruginosa was not influenced by the presence of macrophages in the absence of IFN-γ and IL-17A (Fig. 1C); (ii) IFN-γ causes reduced bacterial killing by macrophage-neutrophil stepwise cocultures (Fig. 2A) but does not directly affect the bactericidal ability of macrophages (Fig. 2B) or neutrophils (Fig. 2C), indicating that macrophage-neutrophil cross talk is required for this effect; (iii) IL-17A slightly promotes the bactericidal activity of macrophage-neutrophil cocultures (Fig. 2B), most likely through a direct effect on neutrophils (Fig. 2C); (iv) neutrophil elastase activity is not affected by the presence of macrophages or cytokines such as IFN-γ and IL-17A (Fig. 3), which suggests that differences in this enzyme during infection probably represent differences in neutrophil numbers rather than increased release per neutrophil; (v) IFN-γ promotes the synthesis of MCP-1, compared to untreated and IL-17A-treated cultures, and reduces the synthesis of IL-1β compared to IL-17A (Fig. 4A); (vi) neutrophils are unlikely contributors to cytokine production, except for IL-8, under these experimental conditions (Fig. 4A; see also Fig. S4 in the supplemental material); (vii) IL-1β production is not affected by elastase activity (Fig. 5B) under these experimental settings; and (viii) IFN-γ and IL-17A differentially regulate IL-1β levels through a direct effect on *IL1B* transcription, which is reduced by IFN-γ and increased by IL-17A (Fig. 6E), rather than by affecting inflammasome activation (Fig. 6A, C, and D and Fig. S3). Hence, this experimental design enabled the analysis of multiple parameters that inform potential outcomes during human antimicrobial responses under different inflammatory conditions.

The overarching aim of this study was to investigate the basis for the positive and negative correlations between IFN-γ and IL-17A production, respectively, and lung function in CF (13). Our original hypothesis was that in the presence of IL-17A, macrophage-neutrophil cocultures will display improved bacteria killing, but the trade-off would be increased release of enzymes that cause tissue damage, such as neutrophil elastase, as described previously in a model of nematode infection (20). This would be in agreement with the pathological role of Th17 cells in chronic inflammation, particularly in CF (13, 14). It was also proposed that IFN-γ would promote neutrophil-mediated killing in the cocultures through exposure of neutrophils to a more proinflammatory cytokine milieu (13) while minimizing tissue damage by reducing the release of neutrophil elastase.

Our findings partly support our original hypotheses and suggest that, in spite of promoting bacterial clearance by neutrophils, IL-17A, compared to IFN-γ, could promote pathology during P. aeruginosa infection through a direct positive effect on IL-1β production. It is also conceivable that during Th17 responses, levels of neutrophil elastase, and the likelihood of tissue damage, will rise following the increase in neutrophil numbers. In contrast, IFN-γ could be beneficial during P. aeruginosa infection by reducing IL-1β (and IL-1α) synthesis and increasing the level of the monocyte chemoattractant MCP-1, rather than promoting neutrophil-mediated bacterial clearance.

The sequential addition of macrophages and neutrophils to bacterial cultures accounted for the initial involvement of macrophages as sensors and triggers of inflammation and neutrophils as efficient and more-abundant microbicidal cells that can increase the likelihood of tissue damage through the release of proteases (1, 2, 21, 22). Macrophages minimally contributed to bacterial clearance, while neutrophils significantly reduced bacterial counts (Fig. 1B and C and Fig. 2). Differences in cell densities (2 × 10^5^ cells/200 µl for macrophage-only cultures and 5 × 10^5^ to 6 × 10^5^ cells/200 µl for cultures of neutrophils with or without macrophages) made direct comparison of the microbicidal capacities of macrophages and neutrophils difficult. Our preliminary results showed that increased cell density leads to increased bacterial phagocytosis by neutrophils, even when the MOI is constant (data not shown).

Our study suggests that IL-17A could potentiate the killing of P. aeruginosa through a direct effect on human neutrophils (Fig. 2). This is the first description of such an effect, which was originally hinted at in the cocultures (Fig. 2B) and clearly observed in neutrophil-only infections (Fig. 2C). This finding is in agreement with a previous study showing increased killing of Streptococcus pneumoniae by IL-17A-treated human neutrophils (23). Further work is required to determine the mechanism(s) responsible. Under steady-state conditions, human neutrophils lack the IL-17RC subunit of the IL-17A receptor (cell surface and mRNA) but express the IL-17RA subunit and the adaptor Act-1 (24, 25). IL-17RC can be induced in human neutrophils by IL-6 and IL-23, and expression can be further increased by Aspergillus fumigatus hyphal extracts in a Dectin-2-dependent manner (25). In addition, IL-17RC expression has been detected in neutrophils from CF patients during exacerbation, and this was linked to the presence of IL-23 in sputum (26). It is possible that IL-17RC expression could also be upregulated in human neutrophils under our experimental settings, i.e., pretreatment with IL-17A for 0.5 h and infection with live P. aeruginosa in the presence of human serum. Future work will involve the analysis of IL-17RC expression during P. aeruginosa infection and investigation of the potential mechanism behind the increased bactericidal activity of IL-17A-treated neutrophils.

In the presence of IFN-γ, macrophage-neutrophil stepwise cocultures displayed a reduced ability to control bacterial growth (Fig. 2B). Since IFN-γ did not directly affect the ability of macrophages (Fig. 2B) or neutrophils (Fig. 2C) to kill P. aeruginosa, these results could be due to IFN-γ-treated macrophages reducing neutrophil activity when in coculture. The mechanism behind this observation is unclear but would be in line with the paradoxical regulatory role described for IFN-γ during inflammation (27–29). IFN-γ-treated macrophages could shorten the life span of neutrophils as a means to control inflammation (30) by promoting apoptosis (31). This would be in agreement with IFN-γ (i) increasing the production of MCP-1 (Fig. 4A and Fig. S3) (13), which promotes monocyte recruitment, a hallmark of resolution of inflammation (2), and (ii) reducing IL-1β and IL-1α, particularly in contrast to IL-17A.

IL-1β is a highly proinflammatory cytokine produced by a two-step process involving the synthesis of pro-IL-1β after the activation of NF-κB and the processing of pro-IL-1β into active IL-1β through the action of inflammasomes (32). P. aeruginosa triggers IL-1β processing through the NLRC4 inflammasome (15). In contrast to other pathogens, early observations demonstrated that IL-1β has a deleterious effect during P. aeruginosa infection: IL-1β deficiency and treatment with IL-1RA were protective against P. aeruginosa pneumonia (33), and IL-1β deficiency was associated with reduced neutrophil recruitment and cytokine production (33). In agreement with data from this study, Cohen and Prince showed that deletion of NLRC4, inhibition of caspase-1, and deletion of IL-1R and IL-18R enhanced bacterial clearance and reduced pathology during P. aeruginosa pneumonia (34). However, the contribution of IL-1β to P. aeruginosa pathogenesis could be dependent on the infection model, as IL-1β has been shown to promote bacterial clearance during corneal infection with P. aeruginosa (18).

It is possible that a neutrophilic inflammatory response could favor chronic P. aeruginosa infection by promoting biofilm formation, which would be detrimental for bacterial clearance (35). Neutrophils have been shown to promote P. aeruginosa biofilm development through the formation of polymers comprised of actin and DNA (35). Results from this study do not support an important role for neutrophil elastase in the generation of IL-1β in the presence of neutrophils (Fig. 5B) under these experimental conditions, which would be in agreement with the constant levels of secreted neutrophil elastase activity detected under all conditions (Fig. 3). Intriguingly, even though human neutrophils have been shown to express key inflammasome components, including NLRC4 (36), no IL-1β was detected in supernatants from neutrophil-only infections. This finding could be due to the short incubation time (1 h). Since NLRC4 can be upregulated by lipopolysaccharide (LPS) in human neutrophils (36), it is conceivable that longer infections could lead to IL-1β production by neutrophils in response to P. aeruginosa. Inflammasome activation and IL-1β secretion in response to Pseudomonas aeruginosa are preserved in CF immune cells (37); therefore, these findings are relevant to the understanding of lung infection and inflammation in this disease.

Inhibition of IL-1β production by IFN-γ has been previously shown *in vitro* (38–41) and during infection with Mycobacterium tuberculosis *in vivo* (42). Mishra et al. (43) showed that pretreatment of mouse macrophages with IFN-γ reduced IL-1β production in response to M. tuberculosis infection by disrupting the NLRP-3 inflammasome through S-nitrosylation. This effect was selective for the NLRP-3 inflammasome, as the activation of AIM-2 and NLRC4 inflammasomes was largely unaffected (43). Our results indicate that the contrasting effects of IFN-γ (reduction) and IL-17A (promotion) on IL-1β production by P. aeruginosa-infected macrophages occur through the regulation of *IL1B* transcription (Fig. 6E) and are in line with previous findings showing inhibition of *IL1B* transcription by IFN-γ in different models (38–41). During P. aeruginosa infection, macrophages treated with IFN-γ or IL-17A displayed comparable levels of LDH release and active caspase-1 upon infection with P. aeruginosa (Fig. 6A, C, and D), suggesting similar capacities to process pro-IL-1β. IFN-γ- and IL-17A-treated macrophages were not significantly different from untreated controls in regard to *IL1B* transcription, but differences between both treatments reached significance (Fig. 6E). These observations indicate that exposure to IFN-γ or IL-17A increases the range of IL-1β production by human macrophages in response to P. aeruginosa infection through a direct effect on pro-IL-1β synthesis rather than pro-IL-1β processing.

IL-1α was detected in supernatants from infected phagocytes albeit in small amounts. IL-1α is the second member of the IL-1 family and, like IL-1β, signals through IL-1R. IL-1α can undergo proteolytic processing in a caspase-1-independent manner, but processing is not required for biological function (44). IL-1α has been implicated in neutrophil recruitment in response to lung infection with P. aeruginosa strain PA103. In agreement with data from this study, IL-1α was produced alongside IL-1β by mouse macrophages infected with PA103 (44). Interestingly, IL-1α release by infected macrophages was highly dependent on the type III effector ExoU, a phospholipase that induces necrotic cell death, and IL-1α contributed to neutrophil recruitment after infection with the wild-type PA103 strain but not when an ExoU-deficient mutant strain was used (44). These results indicate that the contribution of IL-1α to inflammation becomes more relevant under conditions of increased cellular damage. The strain employed in this study (PAO1) lacks the *exoU* gene (45), which would account for the relatively low levels of IL-1α detected in the infection assays (Fig. 4A and Fig. 5C), particularly in comparison with IL-1β. Also, because ExoU can inhibit the NLRC4 inflammasome and caspase-1, the level of IL-1β production would be expected to be higher after infection with PAO1 than after infection with PA103.

This experimental system, macrophage-neutrophil stepwise cocultures, provided novel information regarding macrophage-neutrophil collaboration for bacterial clearance and inflammatory potential under different inflammatory conditions, but as with all *in vitro* systems, it has limitations. For instance, it fails to account for phenotypic changes in neutrophils after extravasation or the massive increase in neutrophil numbers normally observed at inflammatory sites (2, 46), particularly during Th17-driven responses. Future developments will include the use of different macrophage-neutrophil ratios, durations of infections, and live imaging to visualize macrophage-neutrophil interactions, including neutrophil recruitment to the infection site. Testing of more-complex stimuli, such as T cell supernatants as well as clinical bacterial isolates, will be of particular interest.

In conclusion, our findings highlight the key roles for IFN-γ and IL-17A in balancing IL-1β and MCP-1 production and modulating neutrophil activity during P. aeruginosa infection. This study found a role for IFN-γ, particularly in contrast to IL-17A, in regulating macrophage-mediated inflammation, leading to reduced neutrophil-mediated killing and IL-1β and IL-1α synthesis and increased MCP-1 levels. The negative impact of IL-17A during P. aeruginosa infection was recently demonstrated (47). These observations support the efficacy of this macrophage-neutrophil stepwise coculture system to model and improve the understanding of human phagocyte cross talk during infection with extracellular bacteria and how it is affected by prevalent inflammatory conditions.

## MATERIALS AND METHODS

### Preparation of human macrophages.

Monocyte-derived macrophages were generated from buffy coats (Blood Transfusion Service, Sheffield, UK). Peripheral blood mononuclear cells (PBMCs) were isolated by density gradient centrifugation using Histopaque-1077 (Sigma-Aldrich, UK). The PBMC layer was collected and washed, and the monocyte fraction (CD14^+^ cells) was obtained by positive selection using human CD14 MicroBeads and LS MACS columns (Miltenyi Biotec, UK), according to the manufacturer’s instructions. Purified monocytes were suspended in RPMI complete medium (RPMI 1640 [Sigma-Aldrich, UK] containing 15% human AB serum [PAA Laboratories Ltd., UK], 2 mM l-glutamine [Sigma-Aldrich, UK], and 10 mM HEPES [Invitrogen, UK]) and 10 ng/ml recombinant human GM-CSF (rhGM-CSF) (premium grade; Miltenyi Biotec, UK) and plated on ultra-low-attachment 24-well flat-bottom plates (Corning Incorporated, USA) in 500 μl RPMI complete medium containing rhGM-CSF at a density of 1.5 × 10^6^ monocytes/ml. On day 3, 500 µl per well of fresh RPMI complete medium containing rhGM-CSF was added. Macrophages were collected at day 7, washed 3 times in serum-free RPMI 1640, counted, and adjusted to densities appropriate for each assay.

### Preparation of human neutrophils.

The study was approved by the University of Nottingham Medical School Research Ethics Committee (reference no. BT17012011) and the Faculty of Medicine and Health Sciences Ethics Committee (reference no. B195-1801). Ethical approval and informed consent were obtained for all individuals prior to sample collection. Venous blood was collected in EDTA vacutainers (BD Diagnostics-Preanalytical Systems, UK). To isolate neutrophils, 12 ml of Histopaque-1077 (Sigma-Aldrich, UK) was added to a 50-ml Falcon tube (BD Biosciences, UK), and 12 ml of Histopaque-1119 (Sigma-Aldrich, UK) was then carefully layered beneath the Histopaque-1077 layer with the aid of a glass Pasteur pipette. Fresh whole blood (12 ml) diluted 1:1 with RPMI 1640 was layered over the Histopaque-1077 layer, and the tube was centrifuged at 700 × *g* with slow acceleration and the brake off for 30 min at room temperature. Neutrophils were collected and washed once with RPMI 1640. Red blood cells were lysed in 0.2% saline (Sigma-Aldrich, UK) at room temperature for 1 min. An equal volume of 1.6% saline was added to the cells to equilibrate the solution, and cells were washed once with RPMI 1640. Two more washes were carried out in the buffer or medium required for the assay. Cytospins were routinely used to assess the purity of neutrophil preparations.

### Macrophage-neutrophil stepwise infection assay.

P. aeruginosa strain PAO1 (Holloway Collection via D. Haas), serogroup O2/O5, was employed in this study. Bacteria from a −80°C stock were streaked onto lysogeny broth (LB) agar and incubated at 37°C overnight. A single colony was seeded the next day into 5 ml X-Vivo 15 medium without phenol red and gentamicin (Lonza) and incubated overnight at 37°C at 200 rpm. The next day, cultures were diluted to an optical density at 600 nm (OD_600_) of 0.01 and incubated for 3 h at 37°C at 200 rpm. Bacteria were collected and washed, and the appropriate inoculum was made up in HBSS complete medium (Hanks’ balanced salt solution with Ca^2+^ and Mg^2+^ without phenol red [Gibco] containing 5% human serum albumin [HSA; Sera Labs]). Bacteria were preopsonized with normal human serum (NHS) for 20 min at 37°C at 8 rpm in a rotary wheel in 10% NHS in HBSS complete medium. The NHS stock was maintained constant for all experiments shown in Fig. 1 to 6. Experiments shown in Fig.S2 and S4 in the supplemental material were performed with a different NHS stock. Macrophages (2 × 10^5^ in HBSS complete medium) were infected with P. aeruginosa at an MOI of 0.5. Infected macrophages were incubated at 37°C at 8 rpm for 2 h; half of the sample was collected for analysis of CFU, and supernatants were frozen for further analysis. Purified neutrophils (5 × 10^5^ in HBSS complete medium, at a macrophage-to-neutrophil ratio of 1:5) were then added and incubated for 30 or 60 min at 37°C at 8 rpm. In instances where macrophage-only conditions were to be analyzed in parallel with macrophage-neutrophil cocultures, the same procedure was followed except that medium only was added at the point of the addition of neutrophils. To test the effects of IFN-γ and IL-17A on infection outcomes, the same procedure as the one described above was followed, except that macrophages were pretreated for 24 h with the cytokine of interest (either rhIFN-γ [10 ng/ml] [catalog no. 285-IF-100; R&D Systems, Inc.] or rhIL-17A/CF [15 ng/ml] [catalog no. 7955-IL-025/CF; R&D Systems, Inc.]) in the presence of GM-CSF on day 6, and macrophage-neutrophil cocultures were incubated for 30 or 60 min. Cytokine and serum concentrations were maintained throughout the experiment. In some instances, inhibitors of neutrophil elastase (elastase inhibitor III) (catalog no. 324745-5; Calbiochem) or a pancaspase inhibitor (Z-VAD-FMK) (catalog no. V116-2MG; Sigma-Aldrich) was added at the same time as the neutrophils at a final concentration of 20 µM. Samples were processed for CFU analysis and preparation of supernatants, as follows. For CFU, half of the sample was diluted 1:10 in distilled water, passed through a syringe, and serially diluted in phosphate-buffered saline (PBS). For supernatants, samples were centrifuged at 300 × *g* for 5 min at 4°C, and supernatants were further cleared by centrifugation at 13,000 rpm for 10 min at 4°C, collected, aliquoted, and stored at −80°C until analysis.

### Neutrophil infection assay.

Mid-log-phase PAO1 stock cultures prepared as described above were diluted to 2 × 10^7^ bacteria/ml in HBSS complete medium. Bacteria were opsonized by incubation for 20 min at 8 rpm on a rotating wheel at 37°C in the presence of 10% NHS in a 2-ml centrifuge tube. Neutrophils (4 × 10^5^/80 µl in HBSS complete medium) were primed with or without the cytokine of interest (either IL-17A [15 ng/ml] or IFN-γ [10 ng/ml], or complete medium only) for 30 min at 37°C at 8 rpm. Primed neutrophils were added to bacteria (MOI = 5) and gently pipetted up and down. HBSS complete medium was added to control tubes (i.e., P. aeruginosa-only cultures) to make up the final volume as was in the test tubes. Additional NHS was added to achieve a final concentration of 10% NHS in all tubes. All samples were incubated at 8 rpm for 30 min at 37°C and analyzed for CFU as described above.

### Neutrophil elastase quantification.

Neutrophil elastase was quantified using a neutrophil elastase activity assay kit (Cayman Chemicals) according to the manufacturer’s protocol. Fluorescence was read in a Fluoroskan Ascent FL instrument (Thermo Labsystems) at excitation and emission wavelengths of 485 nm and 525 nm, respectively.

### Cytokine quantification.

Culture supernatants were tested for the presence of cytokines using a magnetic Luminex screening kit (catalog no. LXSAHM; R&D Systems, Inc.) according to the manufacturer’s procedure. All samples were read using a Bio-Rad Bio-Plex 200 system.

### Analysis of lactate dehydrogenase activity.

The release of LDH was quantified using an LDH cytotoxicity detection kit (Roche). An LDH positive control (Abcam) with a known concentration of LDH (100 μg/ml) was also used alongside the test samples. Assays under each condition were performed in triplicate wells. The assay was read using a microplate reader (Multiskan FC; Thermo Scientific) at 450 nm and with wavelength correction at 600 nm. The LDH activity in test samples was estimated as [(average OD of test sample)/(average OD of positive control × dilution factor)] × (LDH of positive control [i.e., 100 μg/ml]).

### Analysis of caspase-1 activation in human macrophages.

Macrophages pretreated with IFN-γ or IL-17A for 24 h were infected for 2 h at 37°C at 8 rpm. At 2 hpi, cultures were incubated with Fam-flica (FAM-YVAD-FMK) (catalog no. 655; Immunochemistry Technologies) according to the manufacturer’s protocol. Samples were incubated for 45 min in the dark, after which 100 μl of the culture was placed for 45 min at 4°C on poly-d-lysine (Sigma-Aldrich)-coated 12-mm-round glass coverslips inserted in 24-well tissue culture plates (Costar). Plates were centrifuged for 10 min at 250 × *g* at 4°C, supernatants were removed, and cells were washed three times with apoptosis wash buffer. After the last wash, DNA was labeled with Hoechst (0.5%, vol/vol) for 5 min at room temperature before being washed again twice as described above. Finally, 450 μl of apoptosis wash buffer and 50 μl of fixative were added to each coverslip, mixed, and incubated for 15 min at 4°C. The fixative was taken off, and samples were washed twice again as described above. Coverslips were mounted using Vectashield (Vector Laboratories, Inc.) and analyzed using a Zeiss 710 confocal microscope. z-stacks were processed using Volocity, and corrected total cell fluorescence (CTCF) was calculated as described previously (48–51), using ImageJ 1.51s (Fiji). In brief, cells were selected using the freehand selection tool, and the area, mean, and integrated density (IntDen) (the product of the area and mean gray value) for every single stack were calculated using ROI Manager. After that, the averages of all z-stacks per cell were calculated. The following equation was used to calculate CTCF: integrated density − (area of selected cell × mean fluorescence of background readings). For background, empty areas with no cells were selected.

### Analysis of *IL1B* transcription by qPCR.

Monocyte-derived macrophages prepared as described above were either left untreated or pretreated on day 6 for 24 h with either IFN-γ or IL-17A in the presence of GM-CSF and infected with P. aeruginosa at an MOI of 0.5 under opsonic conditions. Cultures were incubated at 37°C at 8 rpm for 30 min and processed for RNA purification as follows. Total RNA was isolated using an RNeasy minikit (catalog no. 74104; Qiagen) with the incorporation of an on-column DNase digestion (catalog no. 79254; Qiagen) step. The RNA was assessed for quantity and purity using a NanoDrop 2000 instrument (Thermo Scientific). Reverse transcription was performed using Moloney murine leukemia virus (M-MLV) (catalog no. M1701; Promega) and random hexamer primers (catalog no. N0446; New England Biolabs) on standardized quantities of an RNA template. A quantitative PCR (qPCR) assay was performed using a StepOne plus instrument (Applied Biosystems) and GoTaq qPCR master mix for dye-based detection (Bryt green dye incorporating a carboxy-X-rhodamine [CXR] reference dye, catalog no. A6002; Promega). *IL1B* expression was calculated against a relative standard curve using StepOne software to generate a normalized quantity mean. Expression was standardized to the reference gene hypoxanthine-guanine phosphoribosyltransferase (HPRT) and normalized to an untreated control. The primer sequences used for qPCR are as follows and were obtained from Eurofins Genomics: HPRT forward primer AAATTCTTTGCTGACCTGCTG, HPRT reverse primer TCCCCTGTTGACTGGTCATT, *IL1B* forward primer TACCTGTCCTGCGTGTTGAA, and *IL1B* reverse primer TCTTTGGGTAATTTTTGGGATCT. The raw data from the qPCR were analyzed automatically by StepOne software (version 2.3). Normalized mean values were obtained from StepOne software for each independent experiment and analyzed with GraphPad Prism.

### Statistical analysis.

Statistical analysis was performed in GraphPad Prism v 6.02/v 7. Significance was calculated by one-way analysis of variance (ANOVA) with Tukey’s posttest or a Kruskal-Wallis test with Dunn’s posttest when comparing more than two groups. Paired two-tailed Student’s *t* test was used for direct comparison between two conditions for which cells from the same donors were used, as indicated in the text and figures.

## Supplementary Material

Supplemental file 1
