# Supplementary material for: Gamma Interferon and Interleukin-17A Differentially Influence the Response of Human Macrophages and Neutrophils to Pseudomonas aeruginosa Infection
Source: Infect Immun. 2019 Jan 24;87(2):e00814-18. doi: 10.1128/IAI.00814-18 (PMC6346128; doi:10.1128/IAI.00814-18)
Supplement: Supplemental file 1 [file f079a2897c3c4e1ed575dddc2593502d_IAI.00814-18-s0001.pdf]

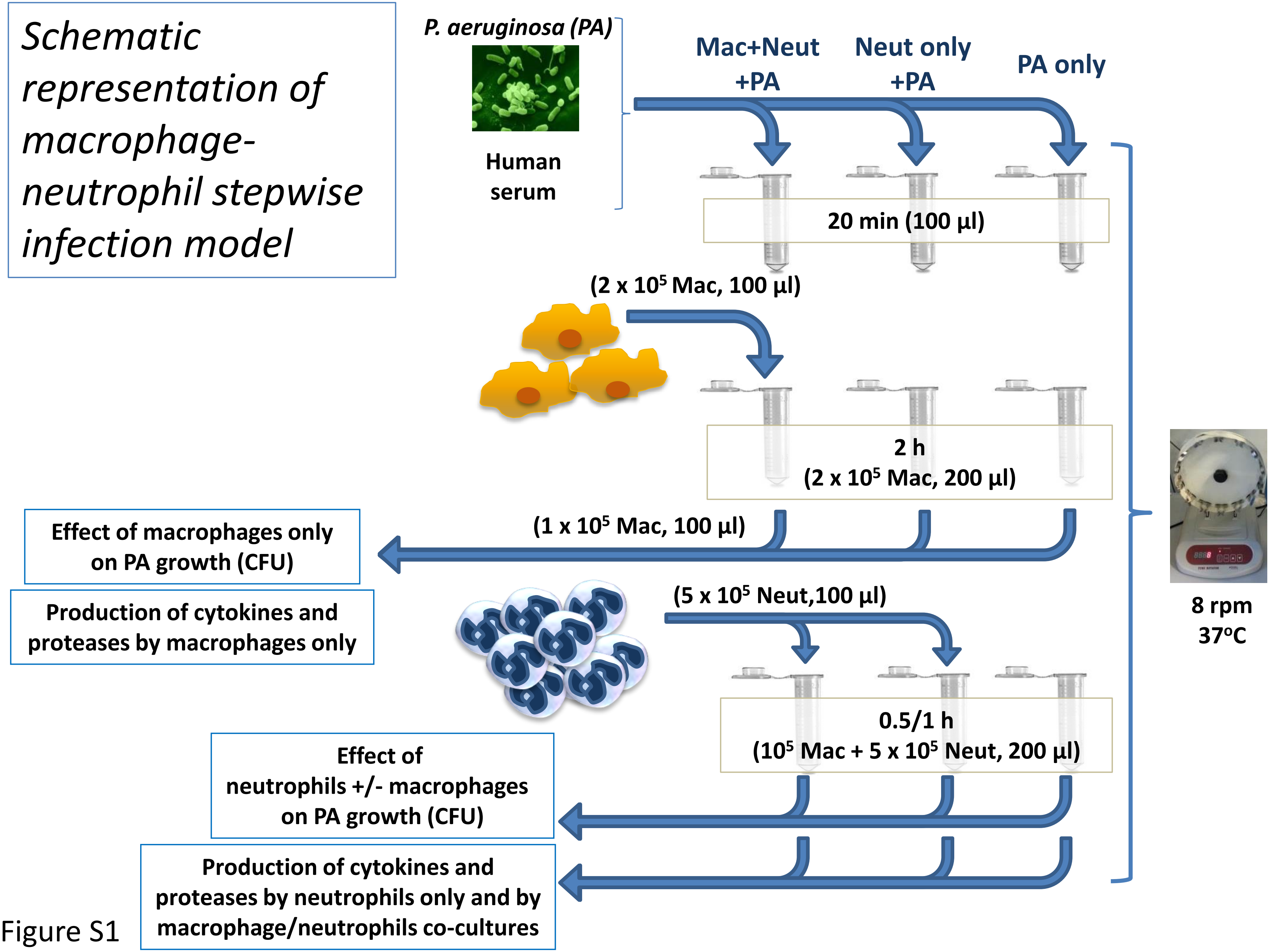
Schematic representation of macrophage-neutrophil stepwise infection model
P. aeruginosa (PA)
Human serum
Mac+Neut +PA
Neut only +PA
PA only
20 min (100 µl)
(2 x 10^5 Mac, 100 µl)
2 h
(2 x 10^5 Mac, 200 µl)
(1 x 10^5 Mac, 100 µl)
Effect of macrophages only on PA growth (CFU)
Production of cytokines and proteases by macrophages only
(5 x 10^5 Neut,100 µl)
0.5/1 h
(10^5 Mac + 5 x 10^5 Neut, 200 µl)
Effect of neutrophils +/- macrophages on PA growth (CFU)
Production of cytokines and proteases by neutrophils only and by macrophage/neutrophils co-cultures
8 rpm
37°C

Figure S1

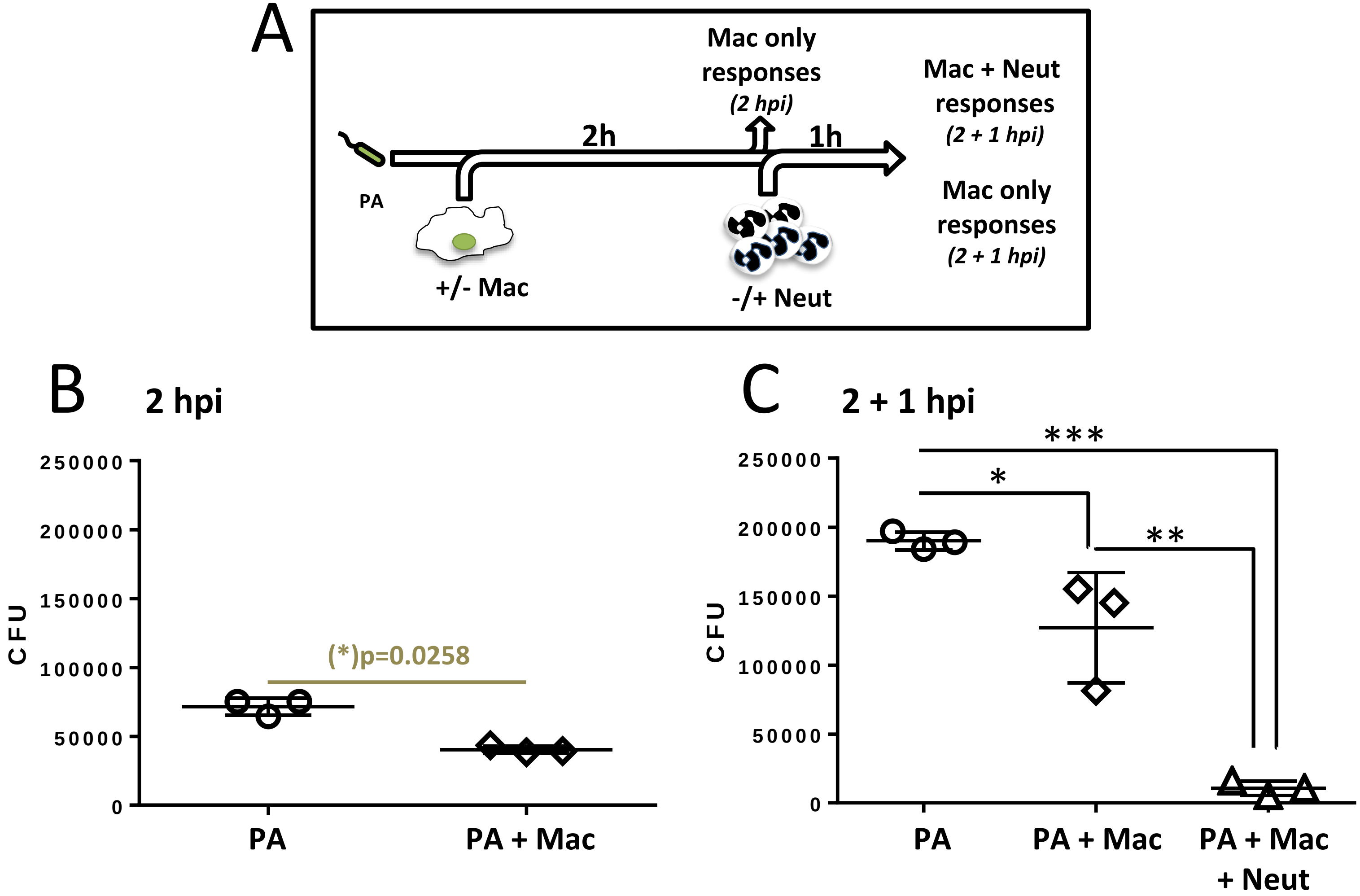

Effect of neutrophils on the bactericidal activity of macrophage-neutrophil stepwise co-cultures.

A. Schematic representation of experimental setting. Human macrophages (2 x 10^5) generated in the presence of GM-CSF were infected with opsonised *P. aeruginosa* as described in materials and methods. At 2 hpi half of the culture was collected and processed for CFU quantification and preparation of supernatants. The second half of the cultures received freshly purified human neutrophils (5 x10^5, macrophage + neutrophil co-cultures, ratio 1:5) or culture media (macrophage only cultures). Cultures were incubated for 1 h and processed for CFU quantification and preparation of supernatants. B. Effect of macrophages on bacterial CFU at 2 hpi. C**.** Contribution of macrophages and macrophages + neutrophils to bacterial CFU at 2 + 1 hpi. PA: *P. aeruginosa*; Mac: macrophages; Neut: Neutrophils. N=3. Black lines: one way ANOVA analysis of PA, PA+Mac, PA+Mac+Neut samples. Grey line: Paired two tailed *t* test analysis.

Figure S2

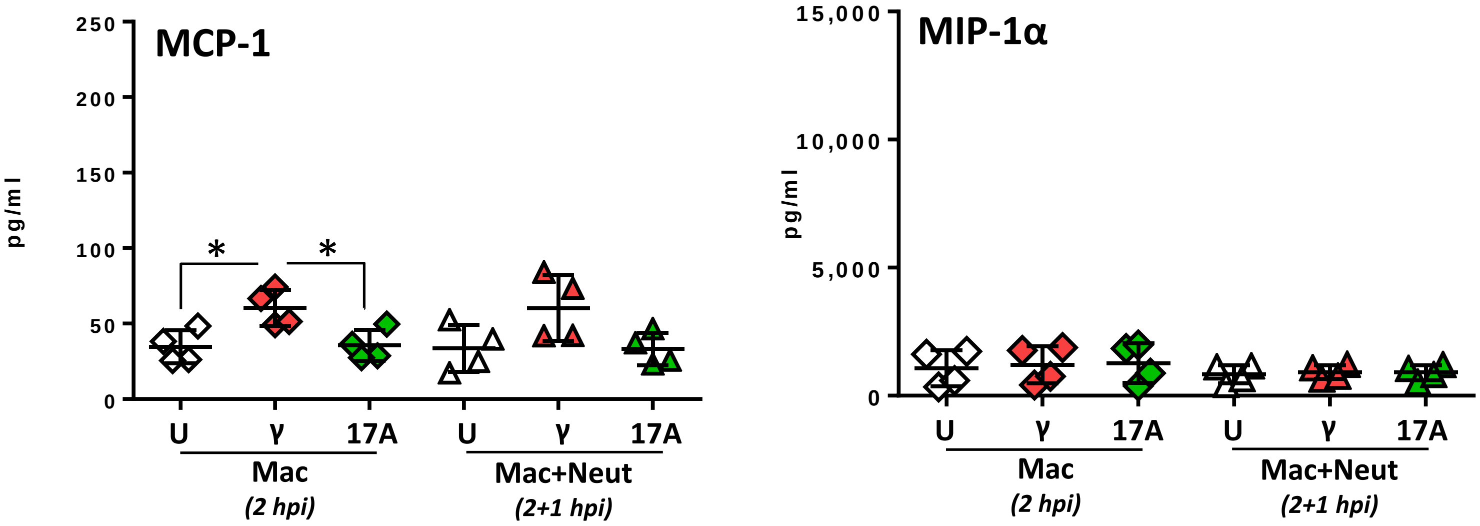

Production of MCP-1 and MIP-1α by uninfected cultures.
Human macrophages (2 x10^5) generated in the presence of GM-CSF were left untreated or treated at day 6 with IFN-γ (γ) or IL-17A (17A). On the following day, macrophages were collected and cultured uninfected in the presence of human serum (2 x 10^5 macrophages). At 2 hpi half of the cultures was collected and processed for preparation of supernatants and freshly purified human neutrophils were added to the macrophage cultures (5 x 10^5 neutrophils, macrophage-neutrophil ratio 1:5). Co-cultures were incubated for 1h and processed for preparation of supernatants.

Figure S3

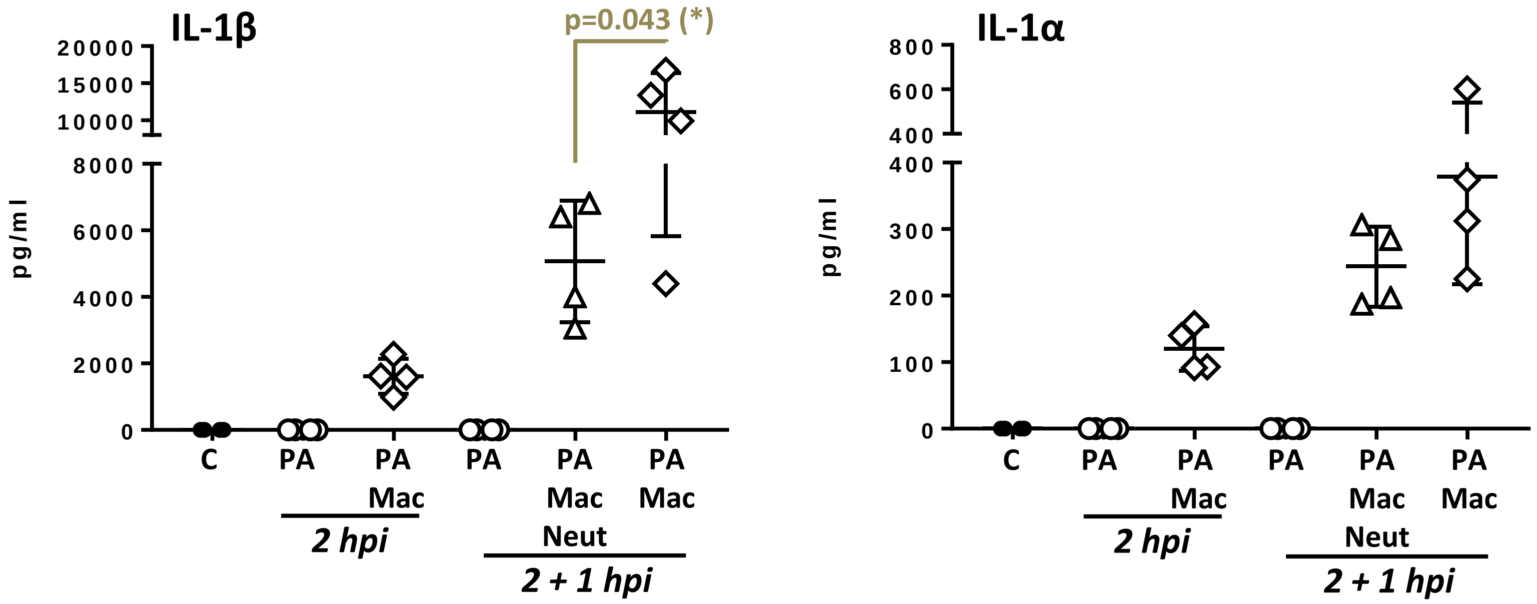

Effect of neutrophils on production of IL-1β and IL-1α by macrophage-neutrophil stepwise co-cultures in response to *P. aeruginosa* infection.
Human macrophages (2 x10^5) generated in the presence of GM-CSF were infected with opsonised *P. aeruginosa* as described in materials and methods. At 2 hpi half of the culture was collected and processed for CFU quantification and preparation of supernatants. The second half of the cultures received freshly purified human neutrophils (5 x10^5, macrophage-neutrophil co-cultures, ratio 1:5) or culture media (macrophage only cultures). Co-cultures were incubated for 1 h and processed for CFU quantification and preparation of supernatants. Cytokines in supernatants were quantified using a Magnetic Luminex Screening kit (Catalogue Number LXSAHM, R&D Systems, Inc.) following the manufacturer's procedure. C: Media only; PA: *P. aeruginosa*; Mac: macrophages; Neut: Neutrophils. N=4. Grey line: Paired two tailed *t* test analysis.

Figure S4A

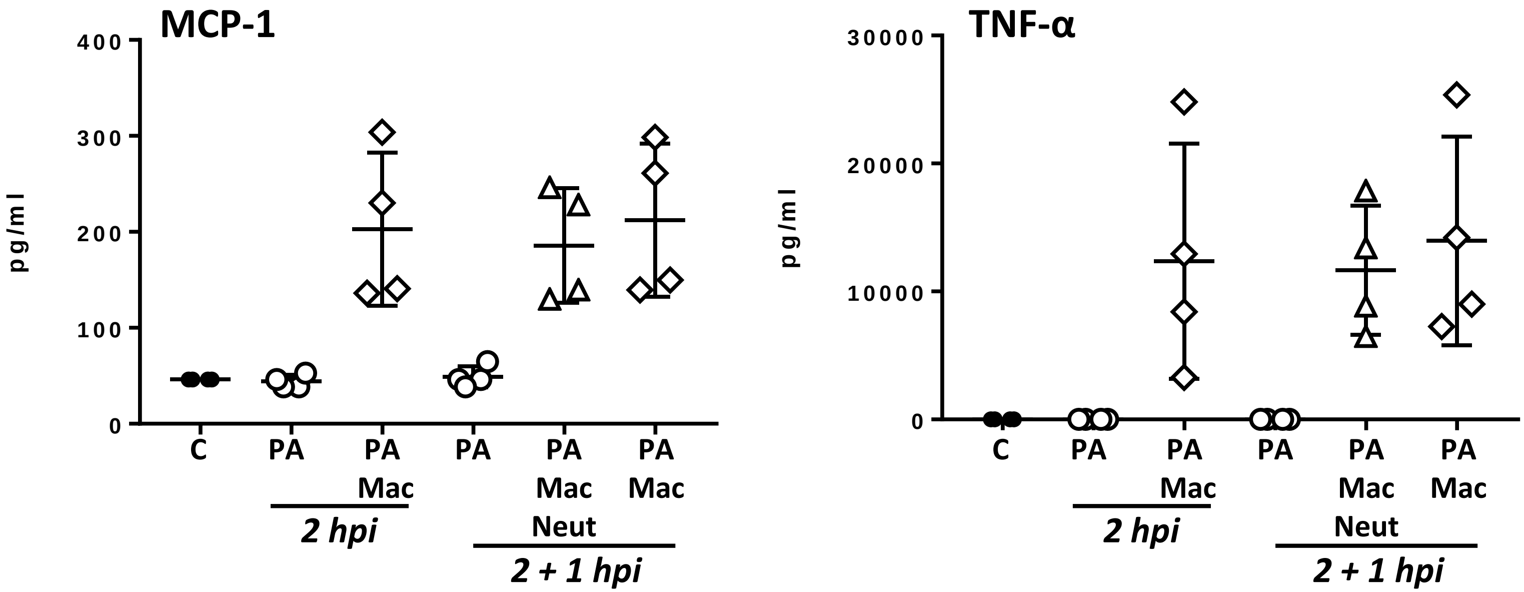

Effect of neutrophils on production of MCP-1 and TNF-α by macrophage-neutrophil stepwise co-cultures in response to *P. aeruginosa* infection.

Human macrophages (2 x10^5) generated in the presence of GM-CSF were infected with opsonised *P. aeruginosa* as described in materials and methods. At 2 hpi half of the culture was collected and processed for CFU quantification and preparation of supernatants. The second half of the cultures received freshly purified human neutrophils (5 x10^5, macrophage-neutrophil co-cultures, ratio 1:5) or culture media (macrophage only cultures). Co-cultures were incubated for 1 h and processed for CFU quantification and preparation of supernatants. Cytokines in supernatants were quantified using a Magnetic Luminex Screening kit (Catalogue Number LXSAHM, R&D Systems, Inc.) following the manufacturer's procedure. C: Media only; PA: *P. aeruginosa*; Mac: macrophages; Neut: Neutrophils. N=4.

Figure S4B

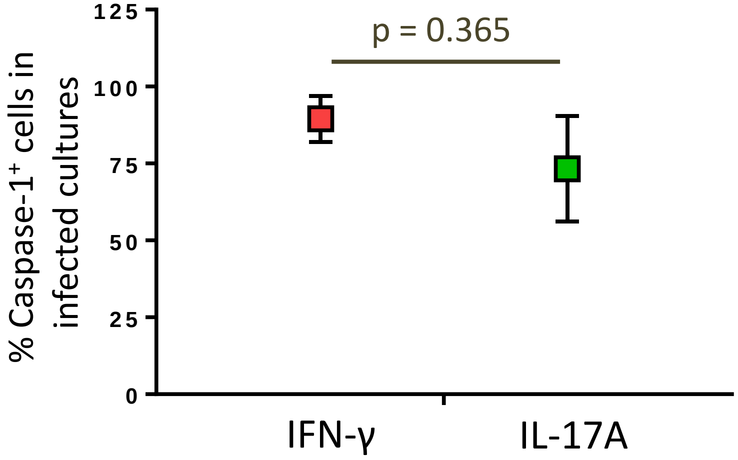

Percentage of *P. aeruginosa*-infected macrophages containing active caspase-1 does not differ between IFN-γ and IL-17A conditions.
IFN-γ- or IL-17A-macrophages were infected with *P. aeruginosa*, treated at 2 hpi with FAM-flica for 0.75 h and processed for confocal analysis. Results derive from samples from 3 independent repeats. Data analysed using a 2-tailed paired *t* test.

Figure S5
